# Rejuvenated Circulating Endothelial Progenitor Cells and Nitric Oxide in Premenopausal Women with Hyperhomocysteinemia

**DOI:** 10.1155/2020/5010243

**Published:** 2020-10-31

**Authors:** Long Peng, Qianlin Gu, Zhenhua Huang, Lijin Zeng, Chang Chu, Xiaoan Yang

**Affiliations:** ^1^Department of Cardiovascular Medicine, The Third Affiliated Hospital, Sun Yat-sen University, Guangzhou 510630, China; ^2^The Division of Emergency Medicine, The First Affiliated Hospital of Sun Yat-sen University, No. 58, Zhongshan 2th Road, Yuexiu District, Guangzhou 510080, China; ^3^Department of Cardiovascular Medicine and Dongguan Cardiovascular Institute, The Third People's Hospital of Dongguan City, Dongguan 523326, China; ^4^Department of Infectious Diseases, The 3rd Affiliated Hospital, Sun Yat-Sen University, No. 600, Tianhe Road, Tianhe District, Guangzhou 510630, China

## Abstract

Hyperhomocysteinemia (HHcy) induced endothelial dysfunction is associated with disturbance in circulating endothelial progenitor cells (EPCs). Nevertheless, whether this unfavorable effect of HHcy on circulating EPCs also exists in premenopausal women is still unknown. Therefore, this leaves an area for the investigation of the difference on the number and activity of circulating EPCs in premenopausal women with hyperhomocysteinemia and its underlying mechanism. The number of circulating EPCs was measured by fluorescence-activated cell sorter analysis, as well as DiI-acLDL and lectin fluorescent staining. The migration and proliferation of circulating were evaluated by the Transwell chamber assay and MTT. Additionally, the endothelial function and levels of nitric oxide (NO), VEGF, and GM-CSF in plasma and culture medium were determined. The number or activity of circulating EPCs and flow-mediated dilatation (FMD) in premenopausal women with or without HHcy were higher than those in postmenopausal women. However, no significant effect of HHcy on the number or activity of circulating EPCs in premenopausal women was observed. A similar alteration in NO level between the four groups was observed. There was a correlation between FMD and the number or activity of EPCs, as well as NO level in plasma or secretion by EPCs. For the first time, our findings illuminated the quantitive or qualitative alterations of circulating EPCs and endothelial function in premenopausal patients with HHcy are preserved, which was associated with retained NO production. The recuperated endothelial repair capacity is possibly the potential mechanism interpreting cardiovascular protection in premenopausal women with HHcy.

## 1. Introduction

Hyperhomocysteinemia (HHcy) induced endothelial dysfunction, accelerating vascular injury in part as a result of atherosclerosis, is one of the independent risk factors for coronary heart diseases (CHD) and other cardiovascular diseases (CVD) [1–5]. Numerous clinical and epidemiological have demonstrated HHcy was significantly associated with flow-mediated dilatation (FMD) reduction, indicting potential vascular endothelial injury [6]. This injury-induced endothelial dysfunction plays a crucial role in the initiation of atherosclerosis. The nature of endothelial dysfunction ultimately represents an imbalance between the magnitude of injury and the repair capacity, increased the peripheral resistance, and further aggravating the endothelial injury, indeed constituted a vicious cycle [5]. Therefore, it is critical to accelerate endothelial repair and maintenance vascular endothelial integrity for the prevention and treatment of CVD by HHcy.

Endothelial progenitor cells (EPCs) are a kind of the primitive cells derived from the bone marrow, which could accelerate reendothelialization, repair the endothelial injury, and improve endothelial function [7–9]. Owing to physiological or pathological factors, the EPCs in the bone marrow will enter the peripheral blood circulation to circulate and proliferate, which can be differentiated into mature endothelial cells, and they participate in vascular repair or formation. The following study shows that the cyclic endothelial cells are involved in repairing damaged endothelial cells, which plays an important role in maintaining the endothelial integrity of the vessel after an arterial injury [10–12]. A growing body of evidence has demonstrated that patients with HHcy have decreased the number of endothelial progenitor cells, increased cell apoptosis rate, and impaired EPCs proliferation and adhesion ability [5, 13]. This phenomenon limited the capacity of EPCs repair and beneficial effect for vascular endothelial.

Clinical and epidemiological have demonstrated that premenopausal women have a decreased prevalence of CVD. However, postmenopausal women have an increased prevalence of cardiovascular diseases, such as congestive heart failure, coronary atherosclerosis, stroke, and arrhythmias [14‐15]. These results indicate that estrogen may play a crucial role in preventing cardiovascular disease. A previous study showed that the activity of circulating EPCs in premenopausal women with prehypertension or diabetes mellitus was preserved [7, 11]. However, further study is needed to determine whether the number and activity of circulating EPCs are still retained in HHcy premenopausal women. Therefore, we hypothesize that the number and functional activity of circulating EPCs in HHcy premenopausal women may be different form postmenopausal women with HHcy. In addition, nitric oxide (NO), vascular endothelial growth factor (VEGF), and granulocyte-macrophage colony-stimulating factor (GM-CSF) plays an important role in regulating mobilization, as well as migration and proliferation of circulating EPCs [16–19].

In this study, we measured the number and functional activity of circulating EPCs in HHcy patients, investigated the level of NO, VEGF, and GM-CSF in plasma and EPCs culture medium, and elucidated the possible mechanism which is responsible for alteration in endothelial repair capacity in HHcy patients.

## 2. Methods

### 2.1. Subject Characteristics

Peripheral blood samples were collected from 80 subjects in our study: twenty healthy premenopausal women, twenty HHcy premenopausal women, twenty healthy postmenopausal women, and twenty HHcy postmenopausal women. The serum homocysteine level was measured by an automatic fluorescence immunoassay method (Abbott, USA). HHcy was defined as a plasma fasting total homocysteine concentration >15 *μ*mol/L [13, 20]. Patients with a history of autoimmune disease, mental disease, diabetes, hypohepatia, renal insufficiency, malignant tumor, gestation period, suckling period, or unwilling to accept the test subjects were excluded. The experimental protocol was approved by the Ethics Committee of our hospitals.  Table 1 shows the baseline characteristics of the four subjects. Peripheral blood samples were used for determining EPCs, blood urea nitrogen, triglycerides, high density lipoprotein cholesterol, LDL-cholesterol, serum creatinine, plasma glucose, high density lipoprotein, total cholesterol, total homocysteine, and so on.

### 2.2. Isolation and Cultivation of EPCs

EPCs were isolated and cultured as previously described [7, 11, 21–24]. In brief, peripheral blood mononuclear cells were obtained from four groups that were isolated by Ficoll density-gradient centrifugation, and it was cultured in endothelial cell growth medium-2 (EGM-2) (500 *μ*mL; Clonetics, San Diego, CA, USA). The cell suspension was incubated at 37°C incubator. After 4 days, we removed nonadherent cells and replaced the medium.

### 2.3. Flow Cytometry

Circulating EPCs were assessed by the number of CD34^+^KDR^+^, peripheral blood mononuclear cells (PBMNCs) by flow cytometry analysis (Beckman Coulter, Fullerton, CA, USA) as previously described [11, 24]. In brief, peripheral blood (100 *μ*L) was incubated with Phycoerythrin (PE) anti-human kinase-insert domain receptor (KDR; 1 : 20; 4A Biotech, Co., Ltd., Beijing, China), fluorescein isothiocyanate (FITC) anti-human CD45 (1 : 10; cat. 4A Biotech, Co., Ltd), and PE-Cy7 anti-human CD34 (1 : 10; 4A Biotech, Co., Ltd). Analysis was done by ACEA NovoCyteTM (ACEA Biosciences, San Diego, CA, USA). The ratio of CD45^−^CD34^+^KDR^+^cells was defined as circulating EPCs.

### 2.4. Double-Positive Fluorescence Identification

After 7 days culture, the plated EPCs on the cell culture flasks were incubated with 1,1′-dioctadecyl-3,3,3′,3′-tetramethylindo-carbocyanine (DiI)-acetylated low density lipoprotein (acLDL) (DiI-acLDL, 10 *μ*L/mL, Molecular probes) at 37°C for 1 h and they were incubated with FITC-labeled Ulex europaeus agglutinin (lectin, 10 *μ*g/mL, Sigma). After staining, the samples were observed under a phase-contrast fluorescence microscope (magnification, ×200). Cell demonstrating double-positive fluorescence were identified as differentiating EPCs.

### 2.5. Migration and Proliferation of EPCs In Vitro

#### 2.5.1. EPCs Migration

EPC migration was determined using a modified Boyden chamber as described in a previous study [22, 23]. In brief, EPCs migration was evaluated by using a transwell chamber (Costar Transwell ® assay, 8 *μ*m pore size, Corning, NY). A total of 2 × 104 EPCs were placed in the upper chamber. The chambers were placed in 24-well culture dish containing 500 *μ*L EBM-2 and human recombinant VEGF (50 ng/mL). After 24 h, EPCs were stained by DAPI. Nonmigratory cells were removed from the upper chamber with the use of an absorbent tip. Cells were migrating into the lower counted by a fluorescence microscope.

#### 2.5.2. EPCs Proliferation

EPCs proliferation was evaluated by 3-(4,5-dimethylthiazol)-2,5-diphenyl tetrazolium (MTT) as described in previous study [22]. In brief, after 7 days of culture, EPCs were digested by 0.25% trypsin and were cultured in serum-free medium in 96-well culture plates for 24 h. Then, EPCs were cultured with MTT (5 g/L; Fluka; Honeywell International, Inc., Shanghai, China) and incubated for a further 4 h. Measurement of EPCs' proliferation by the optical density value at 490 nm.

### 2.6. Measurement of NO, GM-CSF, and VEGF Levels in Plasma and Secretion by EPCs

NO, VEGF, and GM-CSF levels in plasma and secretion by EPCs were evaluated as we previously described [7, 11].

### 2.7. Endothelial Function Evaluation

As described previously, FMD measurement in the brachial artery was performed with subjects in the supine position for the evaluation of endothelial function. Brachial artery diameter was imaged with a 5–12-MHz linear transducer on an HDI 5000 system (Washington, USA). The brachial artery diameters at baseline (*D*0) and after reactive hyperemia (*D*1) was recorded. The FMD [(*D*1–*D*0)/*D*0 × 100%] was used as a measure of endothelium-dependent vasodilation. Pressure in an upper-forearm sphygmomanometer cuff was raised to 250 mmHg for 5 min and FMD calculated as the percentage increase in mean diastolic diameter after reactive hyperemia 55 to 65 s after deflation to baseline. After a further 15 min, 400 *μ*g sublingual glyceryl trinitrate (GTN) was given and diastolic diameter was remeasured after 5 min for measurement of endothelial-independent dilatation.

### 2.8. Statistic Analysis

The statistic software was SPSS V22.0 (SPSS Inc., Chicago, Illinois). All the data were presented as mean values ± SD. Comparisons between the four groups were analyzed by two-factor analysis of variance (premenopausal and postmenopausal women, status of no-HHcy or HHcy). When indicated by a significant F-value, a post hoc test using the Newman-Keuls method identified significant differences among mean values. Univariate correlations were calculated using Pearson's coefficient (*r*). Statistical significance was assumed if a null hypothesis could be rejected at *P* < 0.05.

## 3. Results

### 3.1. Subject Characteristics

As Table 1 shows, all of the subjects had enrolled 80 volunteers. In the baseline values, there was no significant difference in terms of BMI between the four groups. Evidently, the homocysteine level in HHcy premenopausal women and HHcy postmenopausal women was significantly higher than that in the control group (*P* < 0.05). Compared with the postmenopausal women group, the level of estradiol in premenopausal women was higher (*P* < 0.05). In addition, the FMD in postmenopausal women was lower than premenopausal women. The FMD in the HHcy group was lower than the control group, (*P* < 0.05), but there was no significant difference in terms of FMD between HHcy premenopausal women and healthy premenopausal women. There was no significant difference in systolic blood pressure, diastolic blood pressure, HDL, Cr, BUN, LDL, TC, TG, AST, ALT, and FPG for four groups (*P* > 0.05).

### 3.2. The Number, Migratory Capacity, and Proliferative Activities of Circulating EPCs

**Figure 1:**
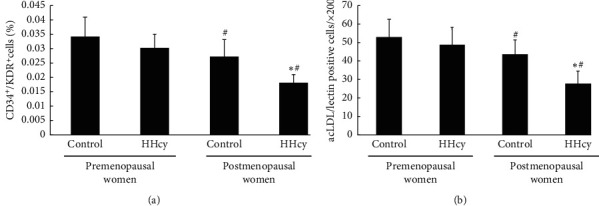
The number of circulating EPCs in the four groups is shown as follows. Evaluated by (a) FACS analysis and (b) phase-contrast fluorescent microscope, the number of circulating EPCs in postmenopausal women was lower than those in premenopausal women. The EPC number in hyperhomocysteinemia in postmenopausal women was lower than that in control postmenopausal women. However, there was no significant difference in the level of the number of circulating EPCs between control and hyperhomocysteinemia premenopausal women. Data are given as mean ± SD. ^☆^vs. control; ^#^ vs. premenopausal women.

The number of circulating EPCs in the four groups is shown in Figure 1. The number of circulating EPCs of circulating EPCs in postmenopausal women was lower than those in premenopausal women. The EPC number in HHcy postmenopausal women was lower than that in control postmenopausal women. However, no significant difference in the level of the number of circulating EPCs between control and HHcy premenopausal women.

**Figure 2:**
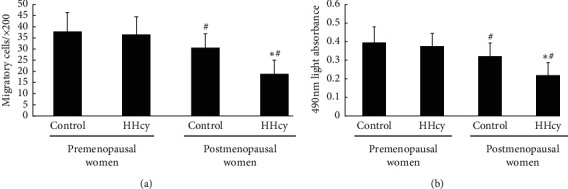
The activity of circulating EPCs in the four groups is shown as follows. The migratory (a) and proliferative (b) activities of circulating EPCs in postmenopausal women were lower than those in premenopausal women. There was no difference in the migratory (a) and proliferative (b) activity between control and hyperhomocysteinemia premenopausal women. Nevertheless, the EPC function in hyperhomocysteinemia postmenopausal women was lower than that in control postmenopausal Women. Data are given as mean ± SD. ^☆^vs. control; ^#^ vs. premenopausal women.

As shown in Figure 2, the migratory (a) and proliferative (b) activities of circulating EPCs in postmenopausal women were lower than those in premenopausal women. There was no difference in the migratory (a) and proliferative (b) activity between control and HHcy premenopausal women. Nevertheless, the EPC function in HHcy postmenopausal women was lower than that in healthy postmenopausal women.

### 3.3. Plasma NO, GM-CSF, and VEGF Levels in Each Group

**Figure 3:**
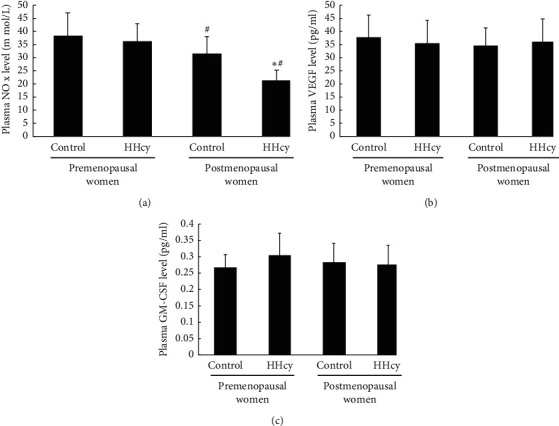
The plasma NO, VEGF, and GM-CSF levels in the four groups were shown as follows. (a) The plasma NO level in postmenopausal women was lower than that in premenopausal women. The plasma NO level in hyperhomocysteinemia postmenopausal Women was lower than that in control, but there was a similarity in control and hyperhomocysteinemia premenopausal women. (b) There was no significant difference in the plasma VEGF level between the four groups. (c) There was no significant difference in the plasma GM-CSF level between the four groups. Data are given as mean ± SD. ^☆^vs. control; ^#^ vs. premenopausal women.

As Figure 3 shows, the plasma NO, VEGF, and GM-CSF levels in the four groups were as follows. (a) The plasma NO level in postmenopausal women was lower than that in premenopausal women. The plasma NO level in HHcy postmenopausal women was lower than that in control postmenopausal women, but there was a similarity in control and HHcy premenopausal women. (b) There was no significant difference in the plasma VEGF level between the four groups. (c) There was no significant difference in the plasma GM-CSF level between the four groups.

### 3.4. NO, GM-CSF, and VEGF Secretion by EPCs in Four Groups

**Figure 4:**
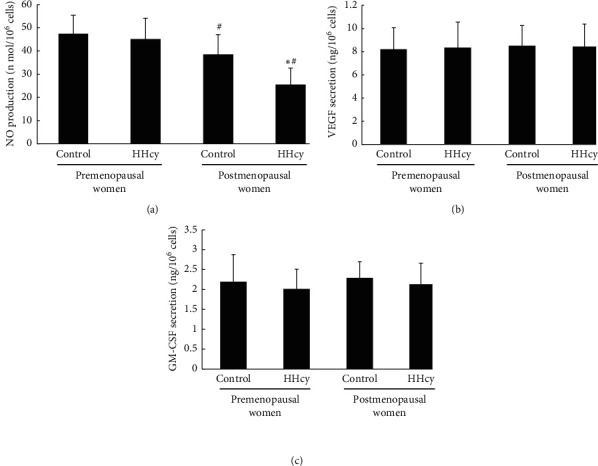
The NO, VEGF, and GM-CSF secretion by EPCs in the four groups was showed as follows. (a) The NO secretion by EPCs in postmenopausal women was lower than that in premenopausal women. No difference in NO secretion by EPCs between control and hyperhomocysteinemia premenopausal women was found. However, the NO secretion level in hyperhomocysteinemia postmenopausal women was lower than that in control. (b) There was no significant difference in VEGF secretion by EPCs between the four groups. (c) There was no significant difference in GM-CSF secretion by EPCs between the four groups. Data are given as mean ± SD. ^☆^vs. control; ^#^ vs. premenopausal women.

As shown in Figure 4. The NO, VEGF, and GM-CSF secretion by EPCs in the four groups was as follows. (a) The NO secretion by EPCs in postmenopausal women was lower than that in HHcy premenopausal women. No difference in NO secretion by EPCs between control and HHcy premenopausal women was found. However, the NO secretion level in HHcy postmenopausal women was lower than that in control premenopausal postmenopausal women. (b) There was no significant difference in VEGF secretion by EPCs between the four groups. (c) There was no significant difference in GM-CSF secretion by EPCs between the four groups.

### 3.5. The Correlation between the Migratory and Proliferative Activities of Circulating EPCs or Plasma NO Level

**Figure 5:**
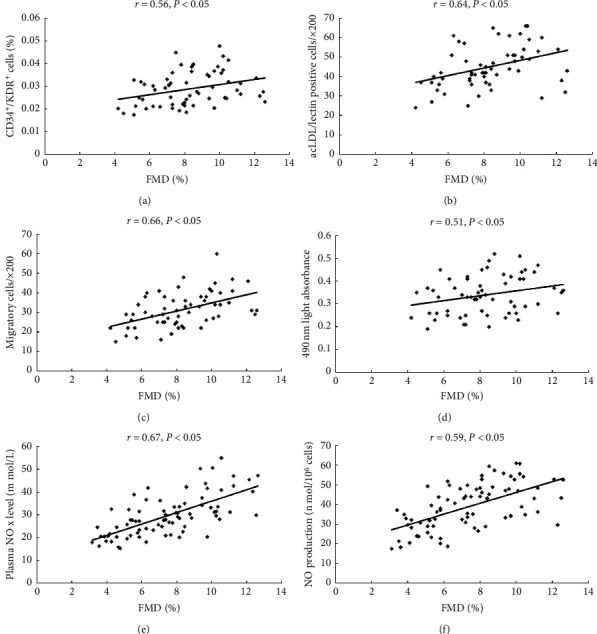
The correlation between circulating EPCs or NO level and FMD was shown as follows. The number of circulating EPCs evaluated by FACS (a) or by cell culture (b) correlated with the FMD. There was a correlation between the EPC proliferation (c) or migratory (d) and FMD. In addition, there was a correlation between the plasma NO level (e) or NO secretion by EPCs (f) and FMD.

As Figure 5 shows, the correlation between circulating EPCs or NO level and FMD was as follows. The number of circulating EPCs evaluated by FACS (a) or by cell culture (b) correlated with the FMD. There was a correlation between EPC proliferation (c) or migratory (d) and FMD. In addition, there was a correlation between the plasma NO level (e) or NO secretion by EPCs (f) and FMD.

## 4. Discussion

In this study, the effect of age difference on the number and activity of circulating EPCs in HHcy women was detected. We found the vascular endothelial function evaluated by FMD in HHcy premenopausal women, as well as the number and activity of circulating EPCs was preserved. Similarly, NO level in plasma or secretion by EPCs in premenopausal women also remained. In addition, we also demonstrated that the number of circulating EPCs, as well as NO in plasma or secretion by EPCs, were significantly reduced, and migratory and proliferative activities of circulating EPCs were also impaired, indicating that the endothelial function-decreased may be closely related to HHcy. There was a significant correction between the number, proliferation, migration of circulating EPCs, and FMD. Similarly, there was a close correction between FMD and NO production in plasma or secretion by EPCS. Therefore, in the present study, which is at least in part associated with the enhanced NO production.

The effects of HHcy on endothelial function have been studied extensively. Accumulating pieces of evidence have shown that HHcy-induced endothelial injury and endothelial dysfunction result in the damage of endothelial integrity, which may accelerate HHcy-related vascular atherosclerosis [13, 25–28]. FMD is a reliable and effective noninvasive new technique [7, 29], and it is widely used to evaluate endothelial dysfunction in CVD. In the current study, we revealed that the FMD in HHcy premenopausal women was preserved. Besides, there is a close correlation between the number and function properties of EPCs and FMD, suggesting increased endothelial repair capacity in premenopausal women. Additionally, we also observed decreased FMD in HHcy men compared with the healthy group, indicating HHcy is a risk factor for vascular endothelial dysfunction.

EPCs can repair injury-induced endothelium [7, 23]. Increasing pieces of evidence have suggested that EPCs contribute up to 25% of endothelial cells in newly formed vessels [13]. The decrease in the number and activity of circulating EPCs may be the related mechanism of endothelial dysfunction and endothelium damage [30]. In our study, we have demonstrated a significant decrease of the number, migratory, and proliferative activities of circulating EPCs in the HHcy postmenopausal women group, but it was preserved in HHcy premenopausal women group, indicating endogenous prevention for endothelial injury in premenopausal women. Therefore, maintaining endothelial integrity is essential for the HHcy postmenopausal women group.

NO not only modulates the mobilization of EPCs from the bone marrow but also improves the function of EPCs. Decreased NO bioavailability by HHcy may reduce nitric oxide-mediated endothelium-dependent vasodilation, which was associated with elevated peroxynitrite in pathological conditions [2]. VEGF and GM-CSF could regulate the number and activity of circulating EPCs [16]. Therefore, we hypothesized that the number and activity of EPCs my be related to NO, GM-CSF, and VEGF. In our study, plasma NO level was restored in HHcy premenopausal women. Besides, plasma NO level in HHcy postmenopausal women was lower than the premenopausal women group, indicating that the decreased number and activity of circulating EPCs may be closely associated with decreased NO production in HHcy postmenopausal women. In addition, a close correction has been observed between NO level in plasma or secretion by EPCs and FMD, indicating NO-mediated prevention of vascular may reverse HHcy-mediated endothelial injury. Furthermore, we discovered the NO secretion by EPCs in HHcy postmenopausal women was lower than healthy postmenopausal women group, indicating inhibited NO production by EPCs may result in a decreased number of circulating EPCs and attenuated activity of EPCs. Decreased NO production secreted by EPCs was a key factor in endothelial dysfunction. The present study indicated that restored exogenous NO production could reverse the number or activity of circulating EPCs in HHcy premenopausal women.

## 5. Limitation

Our research had a few limitations. There were no enough subjects included in this study. In order to reveal whether HHcy can affect the number and function of endothelial progenitor cells in premenopausal women, more research subjects need to be included. Each experimental group should receive more biochemical tests, such as serum insulin and C-peptide, to rule out the influence of confounding factors.

## 6. Conclusions

In conclusion, compared with previous researches, the present study for the first time demonstrated that there exists the effect of estradiol on circulating EPCs in the HHcy group, and the number, migratory, and proliferative activities of circulating EPCs in HHcy premenopausal women are preserved, which may be related with enhanced NO production. In addition, we observed the number and activity of circulating EPCs in HHcy postmenopausal women were attenuated, indicating the decreased endogenous endothelial repair capacity may be the important underlying mechanism accounting for vascular impair which contribute to augment MACE. Therefore, our study provides new insight that increasing the number of circulating EPCs and improving the function of circulating EPCs; meanwhile, enhancing NO production will be a potential target for reversing HHcy-related vascular injury.

## Figures and Tables

**Table 1:** Clinical and biochemical characteristics.

Characteristics		Premenopausal women		Premenopausal women	
		Control (*n* = 20)	HHcy (*n* = 20)	Control (*n* = 20)	HHcy (*n* = 20)
Age (years)		46.1 ± 4.3	44.3 ± 4.8	55.3 ± 4.9^#^	56.7 ± 4.4^#^
Height (cm)		161.2 ± 5.5	160.3 ± 5.1	167.9 ± 4.6^#^	166.6 ± 5.5^#^
Weight (kg)		62.1 ± 5.4	60.0 ± 5.3	64.7 ± 5.5	65.3 ± 4.5^#^
BMI (kg/cm^2^)		23.9 ± 2.1	23.4 ± 2.1	22.9 ± 1.7	23.6 ± 2.0
Systolic blood pressure (mmHg)		117.9 ± 10.4	119.9 ± 8.1	121.7 ± 10.8	122.3 ± 11.2
Diastolic blood pressure (mmHg)		75.9 ± 10.0	72.9 ± 11.1	76.6 ± 8.4	74.1 ± 8.3
AST (mmol/L)		26.0 ± 5.6	26.1 ± 5.6	23.5 ± 5.6	24.3 ± 6.2
ALT (mmol/L)		23.4 ± 4.8	23.0 ± 6.9	20.5 ± 5.4	22.6 ± 5.5
BUN (mmol/L)		5.4 ± 0.9	5.5 ± 1.1	5.1 ± 1.1	5.5 ± 1.0
Cr (mmol/L)		68.9 ± 12.4	71.3 ± 14.7	67.1 ± 16.5	72.4 ± 16.5
LDL (mmol/L)		2.74 ± 0.24	2.86 ± 0.25	2.68 ± 0.41	2.79 ± 0.35
TC (mmol/L)		4.65 ± 0.35	4.80 ± 0.28	4.57 ± 0.51	4.71 ± 0.37
HDL (mmol/L)		1.41 ± 0.25	1.37 ± 0.20	1.44 ± 0.22	1.40 ± 0.15
TG (mmol/L)		1.38 ± 0.18	1.43 ± 0.17	1.36 ± 0.19	1.41 ± 0.14
FPG (mmol/L)		4.58 ± 0.65	4.77 ± 0.64	4.35 ± 0.52	4.63 ± 0.69
Homocysteine (*μ*mol/L)		10.4 ± 1.6	20.7 ± 3 4^☆^	9.4 ± 1.8	21.8 ± 4.5^☆^
Estradiol (pmol/L)		209.2 ± 20.7	202.4 ± 29.8	99.6 ± 16.9^#^	107.5 ± 16.7^#^
FMD (%)		9.46 ± 1.33	8.39 ± 1.22^☆^	8.25 ± 1.07^#^	5.09 ± 0.92^#☆^

Abbreviation: BMI: body mass index; AST: aspartate amino transferals; ALT: alanine transaminase; BUN: blood urea nitrogen; Cr: serum creatinine; LDL: low density lipoprotein; TC: total cholesterol; HDL: high density lipoprotein; TG: triglyceride; FPG: fasting plasma glucose; FMD: flow-mediated brachial artery dilatation. Notes: Data are given as mean ± SD. ^☆^vs. normal weight; ^#^ vs. premenopausal women.

## Data Availability

The data used to support the findings of this study are available from the corresponding author.
